# Salmonella Aortitis Causing a Contained Rupture of a Mycotic Infrarenal Abdominal Aortic Aneurysm Successfully Managed With Endovascular Repair

**DOI:** 10.7759/cureus.114759

**Published:** 2026-08-18

**Authors:** Jordan Scott, Ruchita Simoes

**Affiliations:** 1 Osteopathy, Liberty University College of Osteopathic Medicine, Lynchburg, USA; 2 Hospital Medicine, Chesapeake Regional Medical Center, Chesapeake, USA

**Keywords:** abdominal aortic aneurysm, endovascular aneurysm repair, infectious aortitis, mycotic aneurysm, salmonella aortitis, salmonella bacteremia

## Abstract

Mycotic abdominal aortic aneurysms are rare but life-threatening vascular infections. We report a 71-year-old woman with metastatic small-cell lung cancer receiving immunotherapy. She presented with *Salmonella *bacteremia and was found to have a 5.4-cm eccentric saccular infrarenal abdominal aortic aneurysm with periaortic gas, inflammatory soft tissue, and a large retroperitoneal hematoma consistent with a contained rupture. Emergent endovascular aneurysm repair (EVAR) was performed. She completed six weeks of intravenous ceftriaxone followed by lifelong suppressive antimicrobial therapy. Follow-up imaging demonstrated a patent graft without endoleak. This case highlights the association between non-typhoidal *Salmonella *bacteremia and infectious aortitis and illustrates the role of EVAR, combined with prolonged antimicrobial therapy, in the management of mycotic abdominal aortic aneurysm with contained rupture.

## Introduction

Mycotic aortic aneurysms are uncommon, clinically aggressive vascular infections associated with substantial morbidity and mortality, particularly when diagnosis is delayed or rupture occurs [1,2]. Although the term “mycotic” historically suggests fungal disease, most mycotic aneurysms are bacterial infections of the arterial wall [3]. Non-typhoidal *Salmonella* species are among the most frequently reported causes of infectious aortitis and mycotic aneurysm formation, particularly in older patients or those with underlying vascular disease and systemic comorbidities [4,5]. Computed tomography angiography (CTA) is central to diagnosis because infected aneurysms may show saccular morphology, periaortic inflammatory soft tissue, periaortic gas, hematoma, or rupture [6]. Management generally requires source control with open or endovascular repair combined with prolonged antimicrobial therapy [1,7].

## Case presentation

A 71-year-old woman with metastatic small-cell lung cancer receiving immunotherapy, chronic obstructive pulmonary disease, peripheral arterial disease, systemic lupus erythematosus, hyperlipidemia, and gastroesophageal reflux disease presented with progressive fatigue, weakness, cough, and dyspnea. Initial laboratory evaluation is summarized in Table 1. Blood cultures grew non-typhoidal *Salmonella* species, and urine culture grew *Escherichia coli*.

**Table 1:** Initial laboratory findings. WBC: White blood cell count; Hgb: Hemoglobin; Hct: Hematocrit; MCV: Mean corpuscular volume; BUN: Blood urea nitrogen; CO₂: Carbon dioxide; eGFR: Estimated glomerular filtration rate.

Laboratory parameter	Patient value	Reference range
Sodium	133 mmol/L	135-145 mmol/L
Potassium	3.5 mmol/L	3.5-5.1 mmol/L
Chloride	102 mmol/L	98-107 mmol/L
Carbon dioxide (CO₂)	20 mmol/L	22-29 mmol/L
Blood urea nitrogen (BUN)	16 mg/dL	7-20 mg/dL
eGFR	>60 mL/min/1.73 m²	≥60 mL/min/1.73 m²
White blood cell count	8.9 × 10³/µL	4.0-11.0 × 10³/µL
Hemoglobin	9.0 g/dL	12.0-16.0 g/dL
Hematocrit	28.30%	36.0-46.0%
Platelet	171 × 10³/µL	150-400 × 10³/µL
MCV	88.7 fL	80.0-100.0 fL

**Figure 1:**
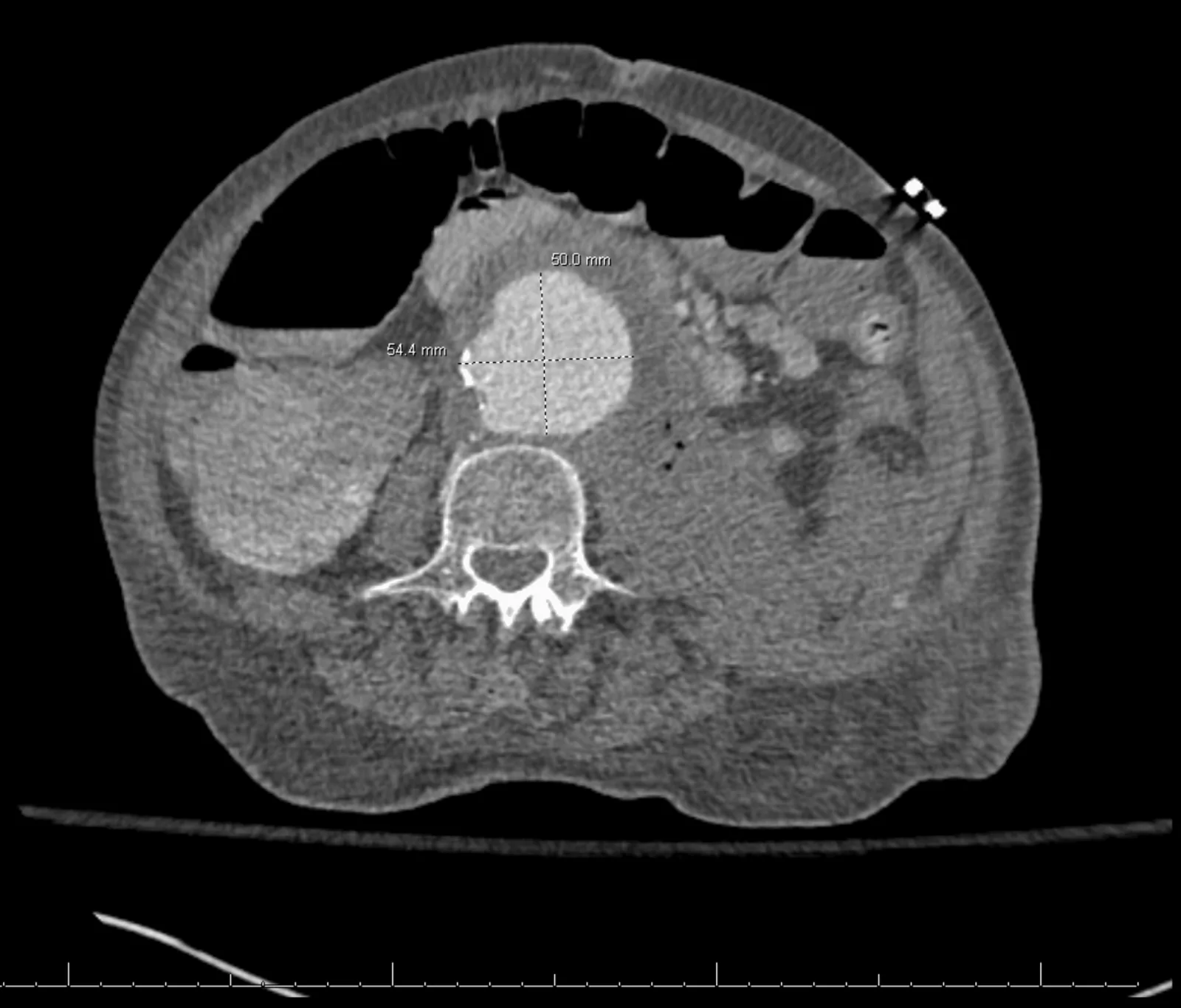
Axial computed tomography angiography (CTA) demonstrating the infrarenal abdominal aortic aneurysm. The aneurysm measures approximately 5.4 × 5.0 cm on the displayed axial image. CTA: Computed tomography angiography.

**Figure 2:**
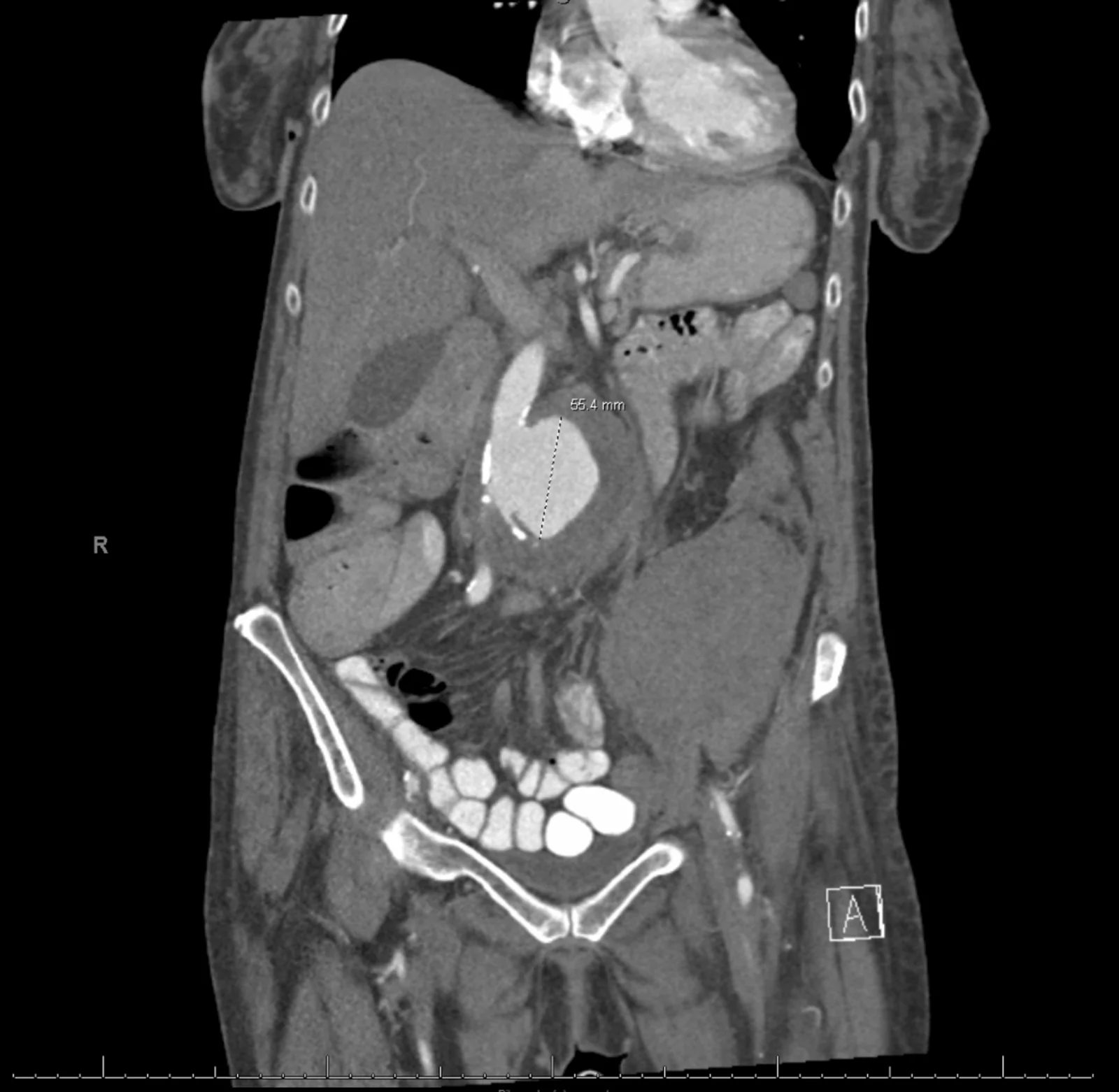
Coronal CTA demonstrating the infrarenal abdominal aortic aneurysm. The coronal image demonstrates the infrarenal abdominal aortic aneurysm, measuring approximately 5.5 cm on the displayed image. CTA: Computed tomography angiography.

During hospitalization, the patient developed worsening left lower extremity claudication. Computed tomography angiography (CTA) of the abdomen and pelvis demonstrated a 5.4-cm eccentric saccular aneurysm arising from the distal infrarenal abdominal aorta and extending into the origin of the left common iliac artery (Figures 1, 2). The aneurysm was associated with extensive periaortic inflammatory soft tissue and multiple foci of periaortic gas. Additionally, there was involvement of the left psoas region and a large left retroperitoneal hematoma extending into the pelvis, findings concerning for a contained rupture. A small splenic infarct was also identified. In the setting of concurrent *Salmonella *bacteremia, the radiographic findings supported a diagnosis of *Salmonella*-associated infectious aortitis with contained rupture of a mycotic infrarenal abdominal aortic aneurysm.

The patient underwent emergent endovascular aneurysm repair (EVAR). Postoperatively, placement of a right internal jugular venous catheter was complicated by a neck hematoma resulting in airway compression and acute hypoxic respiratory failure requiring endotracheal intubation and intensive care unit admission. She was successfully extubated following stabilization.

Serial blood cultures became negative following antimicrobial therapy. She initially received broad-spectrum intravenous antibiotics and was subsequently transitioned to ceftriaxone 2 g daily. Her implanted venous access port was removed during hospitalization. Because infected periaortic tissue remained *in situ* following endovascular exclusion of the aneurysm, the infectious diseases service recommended six weeks of intravenous ceftriaxone followed by lifelong oral suppressive antimicrobial therapy. The patient was discharged home in stable condition after a 12-day hospitalization.

Follow-up CTA approximately seven weeks after EVAR demonstrated a patent infrarenal aortobiiliac endovascular stent graft without endoleak. The abdominal aorta was otherwise normal in caliber, although the follow-up CTA report did not include a quantitative measurement of the excluded aneurysm sac. The left retroperitoneal fluid collection demonstrated an interval decrease in size and greater organization compared with the initial examination. Previously identified splenic infarcts had decreased in size, and a stable 6-mm juxtarenal saccular aneurysm remained unchanged.

## Discussion

This case demonstrates a classic but uncommon vascular complication of non-typhoidal *Salmonella* bacteremia. *Salmonella* species have a recognized ability to cause infectious aortitis, and prior reviews have emphasized the abdominal aorta as a frequent site of involvement [4]. In patients with non-typhoidal *Salmonella* infection, older age, systemic lupus erythematosus, immunodeficiency, and extraintestinal infection have been associated with bacteremia or adverse outcomes [5]. This patient had several relevant risk factors, including advanced age, peripheral arterial disease, malignancy, systemic lupus erythematosus, and *Salmonella* bacteremia.

The CTA findings were highly suggestive of a mycotic aneurysm. Imaging features described in infected aneurysms include lobulated or saccular morphology, irregular arterial wall appearance, perianeurysmal edema, soft-tissue mass, and, less commonly, periaortic gas [6]. In this case, the presence of a saccular infrarenal aneurysm, periaortic soft tissue, gas, psoas involvement, and retroperitoneal hematoma in the setting of *Salmonella* bacteremia favored infectious aortitis with contained rupture rather than a purely degenerative aneurysm.

Open surgical excision and debridement have historically been considered definitive treatment for infected aortic aneurysms, but EVAR has become an important option in selected high-risk patients because it can rapidly exclude the aneurysm and control hemorrhage [1,7,8]. Large multicenter and systematic review data suggest that EVAR can be feasible for mycotic aortic aneurysms, although late infection-related complications remain clinically important [7,8]. Because EVAR leaves prosthetic material and potentially infected periaortic tissue in place, prolonged antimicrobial therapy and close imaging follow-up are essential [1,7,9].

The follow-up CTA in this patient showed no endoleak and interval organization and reduction of the retroperitoneal collection, supporting short-term technical success. However, the residual thick-walled collection was reported as indeterminate for infection. This distinction is important: the case supports *Salmonella*-associated infectious aortitis and contained rupture based on bacteremia and initial CTA findings, but persistent infection of the residual collection cannot be assumed from imaging alone. Long-term suppressive antimicrobial therapy and surveillance were therefore clinically appropriate.

## Conclusions

*Salmonella* bacteremia should prompt consideration of infectious aortitis in patients with vascular risk factors or compatible imaging findings. This case describes a 71-year-old woman with *Salmonella*-associated mycotic infrarenal abdominal aortic aneurysm complicated by contained rupture and successfully managed in the short term with EVAR, prolonged intravenous ceftriaxone, and planned lifelong suppressive antimicrobial therapy. Ongoing surveillance is essential because infected periaortic tissue and prosthetic graft material may remain after endovascular repair.
